# Isolation and identification of local white-rot fungi from West Sumatra and their potential for biodelignification of ruminant feed

**DOI:** 10.5455/javar.2025.l920

**Published:** 2025-06-02

**Authors:** Eli Ratni, Lendrawati Lendrawati, Fadilla Hefzi, Mufidhatul Muqarramah

**Affiliations:** 1 Department of Animal Production Technology, Faculty of Animal Science, Universitas Andalas, Padang, Indonesia.; 2 Department of Biology, Faculty of Mathematics and Natural Sciences, Universitas Andalas, Padang, Indonesia.

**Keywords:** Biodelignification, feed, fungi, lignin, ligninolytic enzyme, ruminant.

## Abstract

**Objective:**

This research aimed to isolate and identify potential white-rot fungi from various locations in West Sumatra, Indonesia, that could be used for biodelignification of animal feed ingredients.

**Materials and Methods:**

Wood samples with fungal infections were directly observed, and the visual method was employed to collect samples. The purified white-rot fungi isolates underwent the Bavendamm test to evaluate their biodelignification capabilities.

**Results:**

Eight of the 15 purified white-rot fungi isolates demonstrated positive results in the Bavendamm test, indicating their potential for biodelignification. Further analysis revealed the presence of three indigenous white-rot fungi species in West Sumatra: *Exidia* sp., *Trametes* sp., and *Phanerochaete* sp. These findings highlight the richness of white-rot fungi biodiversity in the region and underscore their suitability for lignin degradation in animal feed ingredients. Additionally, the successful isolation and identification of these fungi represent a crucial step toward sustainable biotechnological applications in livestock farming.

**Conclusion:**

The identified white-rot fungi have shown promising capabilities for lignin degradation in animal feed ingredients. However, further research is essential to ascertain the optimal enzyme ratio for lignin degradation and to enhance the identification techniques for a broader range of white-rot fungi species. This study provides a foundational step toward improving animal feed quality in the region, especially for ruminants.

## Introduction

The main feed ingredients for ruminants, such as cattle, buffalo, goats, and sheep, generally come from plants that contain lignin. Lignin is an organic polymer compound that binds cellulose and hemicellulose, which are sources of carbohydrates. Lignin cannot be degraded in the rumen of ruminants, so the bound cellulose and hemicellulose cannot be absorbed in the digestive tract [1,2]. The latter types of feed resources are often considered deficient due to their poor digestibility. It results in low nutritional quality in ruminant feed and low performance related to livestock growth and milk production.

Feeding high-fiber animal feed ingredients, such as straw, bagasse, fresh elephant grass, oil palm fronds, and others, is still often done on ruminant farms in Indonesia, including West Sumatra. Thakur et al. [3] reported that fiber content in hardwood stems consisted of cellulose (40%–55%), hemicellulose (24%–40%), and lignin (18%–25%), while softwood stems consisted of cellulose (about 45%–50%), hemicellulose (about 25%–35%), and lignin (about 25%–35%). The presence of lignin in roughages will affect the digestive process of livestock because it is not degraded in the gastrointestinal tract of ruminants and is linked to hemicellulose and cellulose. Therefore, it is necessary to degrade lignin before feed is given to livestock [4].

Physical, chemical, physicochemical, and biological pre-treatment methods have been used to degrade lignin, but each has advantages and disadvantages [5]. The shredding process for feedstuffs requires high energy and expensive machinery. In addition, chemicals can adversely affect the environment, livestock, and farmers due to the toxic waste produced [6,7]. Therefore, biological methods (biodelignification) were chosen as a solution to degrade lignin using microorganisms [8]. This method is more environmentally friendly and minimizes the effects of chemical residues in animal feed. These microorganisms release enzymes that target and break down polymers in lignocellulosic materials [9]. The microorganisms that have the highest potential as lignin degraders are white-rot fungi [4,10].

Enzymes such as lignin peroxidase (LiP), manganese-dependent peroxidase triplicate (MnP), and laccase (Lac), which can mineralize lignin and phenolic organic substrates, are secreted by white-rot fungi, which are members of the Basidiomycota group and are effective at degrading lignin [11]. Weng et al. [12] reported that white-rot fungi can completely break down the lignin into water (H₂O) and carbon dioxide (CO₂). The lignin degradation process will leave decayed wood with a whitish color and fibrous texture [13]. White-rot fungi vary widely in their degree of delignification, based on the species of fungi and the amount of carbohydrates in the lignocellulosic feedstock.

White-rot fungi, which are abundant in tropical countries like Indonesia, including West Sumatra, exhibit diverse types. Despite the recognized importance of bio-delignification in improving the nutritional quality of ruminant feed, there remains a notable gap in the literature regarding identifying and utilizing local white-rot fungi species for this purpose. While white-rot fungi have been extensively studied for their lignin-degrading capabilities, limited research focuses on isolates from specific geographical regions, such as West Sumatra. Furthermore, the optimal enzyme ratios for efficient lignin degradation in ruminant feed have yet to be determined, necessitating further investigation. Thus, a critical need exists to bridge this gap by conducting comprehensive studies to identify indigenous white-rot fungi strains and elucidate their potential for biodelignification in ruminant feed formulations, thereby addressing the challenges associated with lignin-rich feed materials in this region. This study aimed to isolate and identify white-rot fungi in several locations in West Sumatra, Indonesia, that can potentially biodelignify animal feed ingredients.

## Materials and Methods

### Ethical approval

This study does not need ethical approval.

### Study period and sample collection

The study was conducted from May to August 2023 at Labor Biota Sumatra, Universitas Andalas, Padang, West Sumatra, Indonesia. The sample collections were conducted using the visual method based on direct observation of wood that had fungal infections. Sampling was done by taking decayed wood tissue in several locations in West Sumatra, namely Pesisir Selatan (PS) Regency, Padang (P) city, and Solok (S) Regency. They have selected the location of fungi-colonized wood sampling due to the difference between high and lowland areas in West Sumatra. Each location collected five wood tissues, so the total number of wood tissues collected was 15. The samples were cleaned, put into paper envelopes, and labeled. Furthermore, they were stored at room temperature until the isolation process.

### Materials

The materials used are decayed wood tissue, PDA (Potato Dextrose Agar) (Merck), antibiotics (kemicetin), 0.1% tannic acid (Merck), distilled H₂O, 70% alcohol ONEMED, and methylene blue SCIENCECompany. The apparatuses used are Petri dishes, ose needles, analytical balances (Aculab), test tubes (Pyrex), autoclaves (Tomy), incubators (Memmert), hot plate stirrers (Corning), micropipettes, tips, volume pipettes, and refrigerators.

### Preparation of PDA medium

A quantity of 39 gm of PDA powder was carefully weighed and placed into an Erlenmeyer flask. Subsequently, 1,000 ml of distilled H₂O was added to the flask. The resulting solution was then heated using a hot plate at a temperature of 274°C while being stirred continuously until it reached a boil. Following this, the media underwent sterilization in an autoclave at a temperature of 121°C for 15 min. Finally, the media should be cooled down to an appropriate temperature before being poured into Petri dishes.

### Fungi isolation

Prior to the isolation process, a volume of 500 ml of PDA media was combined with two antibiotic capsules (kemicetin), aiming to safeguard against bacterial contamination. To prevent airborne contamination, the PDA media was carefully poured into sterile Petri dishes within a controlled laminar airflow environment. The media were allowed sufficient time to solidify and achieve firmness. Subsequently, wooden sections infected by fungi were sectioned into square pieces and subjected to washing using distilled H₂O. These pieces were then dried on absorbent paper towels. Following this drying phase, they were implanted onto the prepared media across three replicates for each wooden sample. The samples underwent incubation under specific laboratory conditions for a duration spanning 3–4 days using designated culture racks. Finally, fungal colonies emerged as evident results from successful growth in these cultures.

### Isolate purification

The white-rot fungi colonies obtained from the media were subjected to purification by transferring them onto a fresh culture medium. Additionally, these purified isolates were preserved as stock cultures to facilitate subsequent testing and experimentation.

### Bavendamm test

The Bavendamm test, also known as the Bavendamm reaction, detects phenolic hydroxyl groups in organic compounds by forming colored azo compounds. The test was first described by Bavendamm in 1928 [14]. It was executed to discern between white-rot and brown-rot fungi in a fungal culture. This differentiation was achieved by introducing 0.1% tannic acid into PDA media. The purified isolates were subsequently cultivated on this treated media and incubated for 5–7 days. It should be noted that if the surface of the media displays a brown hue during this incubation period, it signifies that the fungus is classified as belonging to the group of white-rot fungi.

### Identification of fungi

The fungal cultures cultivated on Bavendamm media were rejuvenated on new PDA media. They were then subjected to an incubation period of 3–4 days. Once colonies developed, they were carefully transferred onto a glass plate using an ose needle. A single drop of methylene blue dye was introduced into the culture for staining purposes. Subsequently, microscope analysis was conducted to examine the hyphae, basidiospores, and any distinctive features exhibited by each fungus species. The identification process involved comparing the observed characteristics with those provided in a fungal identification reference book to ascertain their respective genus.

## Results and Discussion

### White-rot fungi isolate

**Figure 1.**
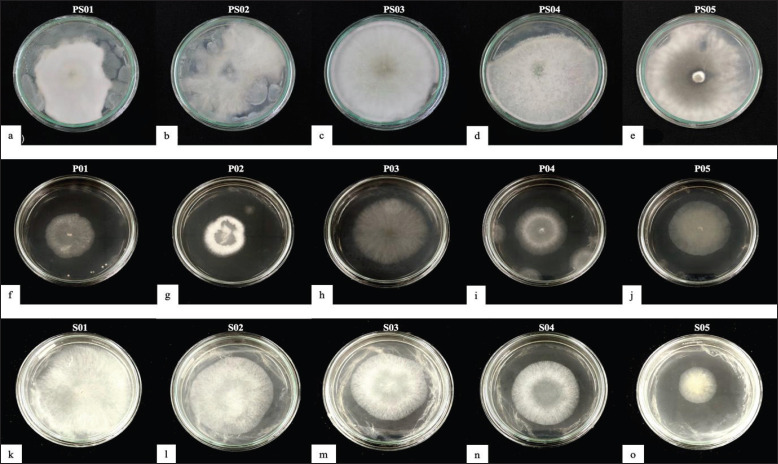
Purified white-rot fungi isolates. a–e = Pesisir Selatan, f–j = Padang, k–o = Solok.

The decayed wood was taken from three locations in West Sumatra. Then, the fungi were isolated using Potato Dextrose Agar (PDA) media. Isolates were incubated for 3–4 days at room temperature. As shown in Figure 1, 15 fungal isolates were successfully purified. These included five isolates located in PS, five isolates from Padang (P), and five isolates from Solok (S). All the isolates showed a white colony color that was like cotton. Peralta et al. [15] reported white-rot fungi growing on live trees or dead wood in temperate to tropical environments. Wood over-grown by this fungus will have a whitish (white) appearance like cotton.

### Bavendamm test

**Figure 2.**
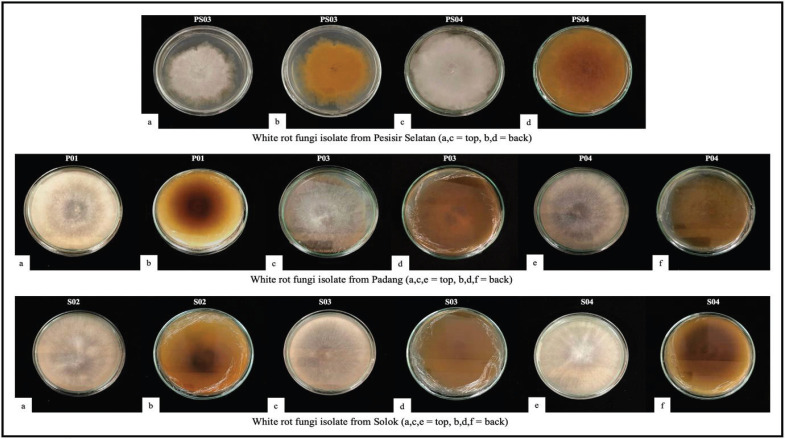
White-rot fungi isolate positive for the Bavendamm test.

The Bavendamm test was used to identify whether the purified fungi were white-rot fungi and to determine the ligninolytic enzyme activity in the fungi. In the Bavendamm test method, each isolate is tested and isolated on a PDA medium with the addition of 0.1% tannic acid as a carbon source [16]. From the 15 isolates that had been purified, eight isolates were positive for the Bavendamm test, consisting of two isolates from PS, three isolates from P, and three isolates from S (Fig. 2). The positive reaction (+) Bavendamm test shows that a light to dark brown color forms in the medium around the colony. This indicates that the fungi can break down tannic acid by releasing extra-cellular oxidase enzymes and are white-rot fungi [17]. Conversely, the Bavendamm test demonstrates a negative reaction (–) when no brown color is observed in the media.

Fungi use the enzyme phenol oxidase, which is crucial for breaking down lignin, to oxidize the phenol in the medium. This is what gives the medium its brown color. White-rot fungi are known to be the most potent lignin degraders, resulting in compounds that dissolve in H₂O and CO₂. Lignin breaks down into CO₂, which gets into wood polysaccharides and protects them with lignin-carbohydrate complexes [18]. White-rot fungi produce ligninolytic extracellular enzymes during the lignin degradation process. Currently, three types of ligninolytic extracellular enzymes are known: laccase (Lac), lignin peroxidase (LiP), and manganese peroxidase (MnP). Lacs are copper-containing enzymes used to oxidize a variety of aromatic hydrogen polyphenols and non-phenolic compounds; LiP degrades non-phenolic components, while MnP can degrade phenolic components of lignin [19,20]. Gáspár et al. [21] reported that organisms with a positive Bavendamm reaction also had a significantly positive Lac test with syringaldazine. Most organisms with a negative test also had negative Lac and peroxidase tests.

White-rot fungi make ligninolytic enzymes as a byproduct of their metabolism because breaking down lignin does not give them energy [22]. Lignolytic enzyme activity can result in high levels of delignification. The best method for biodelignifying lignocellulose biomass is to combine two or three ligninolytic enzymes; this is more efficient than using microbial cell biodelignification or individual enzymatic biodelignification. Ahmad et al. [23] reported the effectiveness of the enzymes LiP, MnP, and Lac for lignin degradation in wheat straw, bagasse, and rice straw. The best delignification rates were reported for wheat straw and bagasse at 58.5% and 55% degradation rates, respectively, with a 2:1:2 MnP:LiP:Lac ratio. At a rate of 52% degradation, rice straw was optimally delignified at a 1:2:2 ratio. Even though applying pure enzymes increases efficacy (by almost two times), doing so is neither economical nor cost-effective, particularly when applying commercial enzymes for large-scale operations involving lignocellulosic substrates derived from nature.

White-rot fungi are also efficient degraders of plant cell wall constituents because of their extracellular enzymes [24]. In ruminants, fiber degradation is adversely affected by lignin, a key cell wall component [25]. White-rot fungi’s capacity to break down lignin positions them as promising choices for converting heavily lignified agricultural biomass into ruminant feed [26]. For animal feed, a higher level of lignin degradation is required to enhance the digestibility of the fermented feed for ruminants [27]. After incubating treated wheat straw for seven weeks, Tuyen et al. [28] observed that three white-rot fungi, *Ceriporiopsis subvesmipora*, *Lentinula edodes*, and *Pleurotus eryngii*, showed a high lignin-to-cellulose loss ratio, increasing the in vitro degradability in the rumen fluid by 20%–60%. Nayan et al. [26] also reported that *Ceriporiopsis subvermispora* has the potential to enhance the ruminal degradability of wheat straw. Meanwhile, Yanti et al. [29] reported that biodelignification with *Pleurotus ostreatus* for 28 days produces the best sugarcane shoots, with 12.83% of lignin decreasing its effectiveness as an alternative for ruminant feed.

### Identification of white-rot fungi

Identification was carried out on eight isolates of fungi that were positive for the Bavendamm test. Identification is done by observing the characteristics of hyphae and spores, which are then compared to the identification book. Based on the identification results, three fungal genera of three families were obtained, namely *Exidia* sp., *Trametes* sp., and *Phanerochaete* sp., shown in Table 1.

**Table 1.** Identification results of white-rot fungi from several locations in West Sumatra.

Isolate	Picture	Characteristics	Family	Genus
PS03	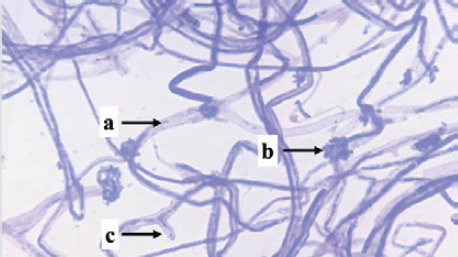 Note: (a) hyphae, (b) spore, (c) clamp connections	Nonseptate hyphae, clustered spores, clamp connections	*Auriculariaceae*	*Exidia sp.*
PS04	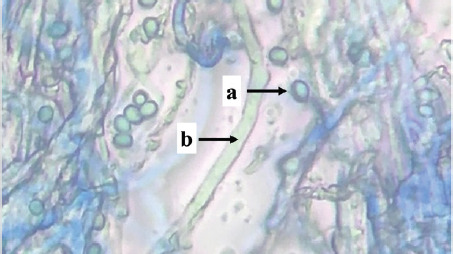 Note: (a) spore, (b) hyphae	Nonseptate hyphae, single spore	*Polyporaceae*	*Trametes* sp.
P01	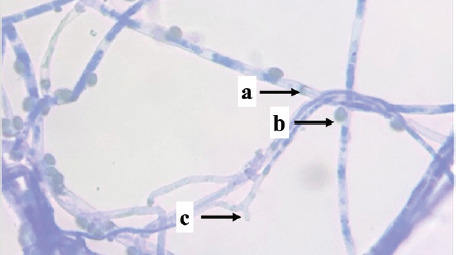 Note: (a) hyphae, (b) spore, (c) clamp connections	Septate hyphae, clamp connections	*Phanerochaetaceae*	*Phanerochaete* sp.
P03	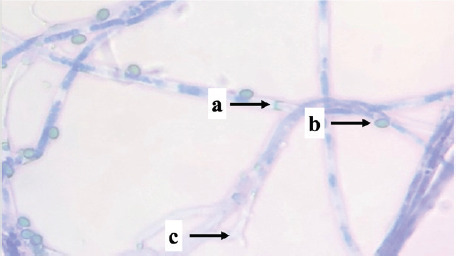 Note: (a) hyphae, (b) spore, (c) clamp connections	Septate hyphae, clamp connections	*Phanerochaetaceae*	*Phanerochaete* sp.
P04	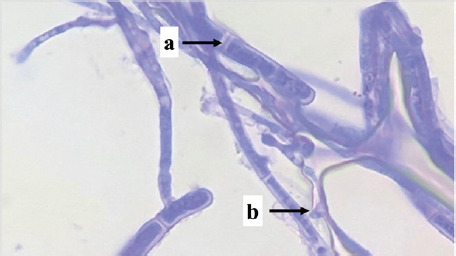 Note: (a) hyphae, (b) clamp connections	Septate hyphae, clamp connections	*Phanerochaetaceae*	*Phanerochaete* sp.
S02	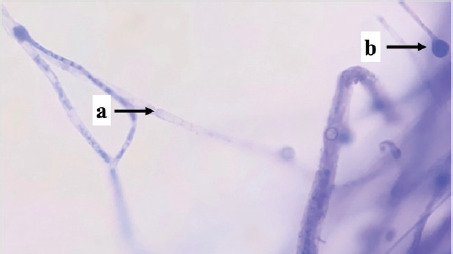 Note: (a) hyphae, (b) conidia	Septate hyphae, round conidia	*Phanerochaetaceae*	*Phanerochaete* sp.
S03	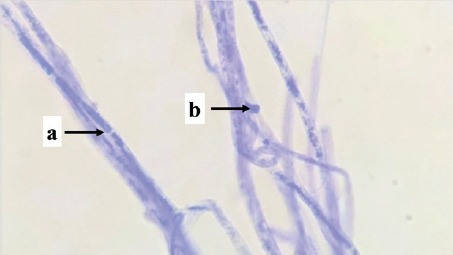 Note: (a) hyphae, (b) conidia	Septate hyphae, round conidia	*Phanerochaetaceae*	*Phanerochaete* sp.
S04	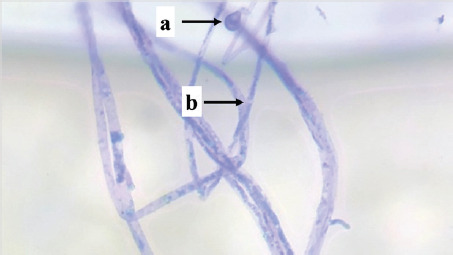 Note: (a) conidia, (b) hyphae	Septate hyphae, round conidia	*Phanerochaetaceae*	*Phanerochaete* sp.

Isolate PS03 is a type of fungus, *Exidia* sp., which is classified in the *Auriculariaceae* family. *Exidia* sp. includes jelly fungi that are saprotrophic in dead wood and are a group of organisms that can break down lignin. There is a clamp connection that plays a role in moving the cell nucleus in the process of hyphal development. *Exidia sp.* can colonize newly dead wood because this fungus destroys tissue from the vascular cambium.

Isolate PS04 is a type of fungus, *Trametes* sp., which is classified in the *Polyporaceae* family. *Trametes* sp. has thick-walled hyphae, has no partitions, has elliptical spores, and has clamp connections. *Polyporaceae* hyphae have clamp connections, and some do not. The hymenium is tubular like a gill (lamella), not thick, and produces 2–4 spores per basidium. This fungus is a white weathering fungus and can produce the enzyme Lac, which helps the fungus degrade lignin.

Isolates P01, P03, P04, S02, S03, and S04 are types of fungi, *Phanerochaete* sp., which are classified in the *Phanerochaetaceae* family. *Phanerochaetaceae* spores are elliptical, thin-walled, clear, and hyphal with a normal lumen, elongated, thick-walled, and not bulging. Zmitrovich et al. [30] reported that *Phanerochaete* sp. has hyphae that are concentrated and have clamp connections, and the spores are produced singly and clustered at the ends of the hyphae. *Phanerochaete chrysosporium* is one of the white-rot fungi that exists in wood and produces extra-cellular enzymes LiP, MnP, and Lac.

## Conclusion

We can conclude that, based on the 15 purified white-rot fungi isolates, eight isolates were positive for the Bavendamm test. Three types of local white-rot fungi of West Sumatra were found: *Exidia* sp., *Tramete*s sp., and *Phanerochaete* sp. More research is needed to find the best enzyme ratio for breaking down lignin in ruminal feeding and to figure out how to identify different types of white-rot fungi.

## List of abbreviations

gm, grams

Lac, laccase

LiP, lignin peroxidase

min, minutes

ml, milliliter

MnP, manganese-dependent peroxidase triplicate

P, Padang city

PDA, potato dextrose agar

PS, Pesisir Selatan regency

S, Solok regency

sp., species.
